# The Performance of Super Absorbent Polymer (SAP) Water-Retaining Asphalt Mixture

**DOI:** 10.3390/ma12121964

**Published:** 2019-06-18

**Authors:** Jiuguang Geng, Mingyuan Chen, Tao Shang, Xun Li, Y. Richard Kim, Dongliang Kuang

**Affiliations:** 1 School of Materials Science and Engineering, Chang’an University, Xi’an 710061, China; gengjiuguang@163.com (J.G.); shangtaoca@163.com (T.S.); muzixun1024@163.com (X.L.); kim@ncsu.edu (Y.R.K.); kuangdl@163.com (D.K.); 2 Engineering Research Center of Ministry of Education for Transportation Pavement Materials, Xi’an 710061, China; 3 Department of Civil, Construction, and Environmental Engineering, North Carolina State University, Campus Box 7908, Raleigh, NC 27695-7908, USA

**Keywords:** SAP mortar, porous asphalt mixture, water-retaining, microstructure

## Abstract

Lowering the temperature of the road surface is one efficient way to alleviate the urban heat island effect. Therefore water-retaining asphalt mixture was produced by adding super absorbent polymer (SAP) containing cement mortar to the porous asphalt mixture. In this study, the water absorption capacity, mechanical strength and fluidity of the cured water-retaining mortar were investigated to determine the optimum SAP dosage in water-retaining mortar. Furthermore, the microstructure of the hardened water-retaining mortar was studied using scanning electron microscopy (SEM) to determine the morphology and distribution of SAP in the final product, which may help to understand the influence of SAP on water retention performance and decipher its underlying mechanism. Compared to the raw porous asphalt mixture, the water-retaining asphalt mixture showed good moisture susceptibility (retained stability (RS) ≥ 88.2%, tensile strength ratio (TSR) ≥ 81.8%), good rutting resistance (9336–10,552 times/mm) and low temperature crack resistance (3383–3621 MPa), as well as significant cooling effects (10–12 °C). The results illustrate that the prepared SAP water-retaining asphalt mixture has good potential in reducing dust and enhancing road performance.

## 1. Introduction

Asphalt pavement is widely used nowadays due to its durability and resilience. The continued absorption of heat radiation from the sun can lead to the heating of the asphalt pavement and creates a high temperature on the pavement surface. This not only affects people’s comfort, but can be detrimental in other ways. It decreases the service life of the pavement and results in huge energy consumption and economic loss. Therefore, the concept of sponge pavement material design was proposed recently [1]. On the basis of the original permeable asphalt pavement with large-pore structure, the super absorbent polymer (SAP) mortar material is poured to fill the pores within the asphalt structure, which enables the pavement to have high water absorption and retention capacities. Meanwhile, part of the pore structure can still be retained inside the paving material, facilitating the discharge of the excess water [2,3]. After precipitation, the moisture absorbed by the road surface can dissipate the heat of the pavement surface by water evaporation. This helps to decrease the temperature of road surfaces and increase environmental humidity [4]. Furthermore, it may also serve to suppress road dust and reduce disease.

In recent years, more and more researchers have begun to study semi-flexible asphalt pavement. Setyawan and his coworkers [5] established a unique correlation between asphalt skeleton, grouting, aggregate type and size by evaluating the compressive strength of semi-flexible pavements, and compared with conventional concrete, only for compressive strength test, but this comparison was one-sided. Pei et al. [6,7] determined that the cement slurry was more suitable as a grouting material by comparing the fluidity, strength and drying shrinkage of cement slurry and cement mortar, and then pouring the two grouting materials into the asphalt mixture for performance evaluation. Ding et al. [8] analyzed the performance of semi-flexible pavement in the volume parameters of matrix asphalt mixture, and found that the high-low temperature stability of semi-flexible pavement materials was superior to ordinary asphalt pavement materials, and the performance of the matrix asphalt mixture with more air voids was better. At the same time, some scholars improved the performance of grouting materials by using additives. Wang et al. [9] added carboxyl latex to cement mortar as an additive to determine the best mix ratio, and then tested the properties of semi-flexible pavement material with carboxyl binder for asphalt mixture matrices with different pore contents (high temperature rutting, low temperature crack, water damage and fatigue). Ke et al. [10] conducted a comprehensive evaluation of the performance of latex cement mortar, and latex cement mortar into the composite sample, effectively improving the road performance of the asphalt mixture, but the latex cement was prone to segregation. For the matrix material of semi-flexible asphalt mixture, Kandhal [11] proposed a new open wear-resistant layer coarse aggregate void index and the best asphalt dosage method. Then, the residual tensile strength (TSR) was tested after the freeze-thaw cycle to test the properties of the mixture. Pei [12] studied the influence of fractal theory on the cavity characteristics of Open Graded Friction Course (OGFC), and quantitatively analyzed the relationship between void ratio and void equivalent diameter and the influence of void distribution on drainage performance. However, the porous asphalt mixture has a low water absorption rate and cannot store water easily. To ameliorate road stability and alleviate urban heat island effects, Tadanobu [13] designed a water-preserving and cooling pavement made of porous asphalt and filler, and analyzed the road surface cooling effect and the improvement of urban temperature environment through experiments and models. Hendel et al. [14] conducted road heat flux observation and analysis. The influence of sprinkling frequency and timing on the cooling effect of the pavement were considered by Santamouris [15], who analyzed the cooling effects of the heat-reflecting coating and water-retaining sidewalk technologies on the pavement. Tianqing [16] developed a flexible pavement with water retention and cooling effects and described its construction method. Researchers from France and Japan [17,18] successfully developed the “grinding and grouting asphalt concrete pavement construction method” and using a mineral material waterproof paving test area, conducted a test of this paving as a heat-resistant pavement. The final test results showed that the ambient temperature of the water-retaining pavement near the surface was significantly lower than that of the permeable pavement, indicating water retention. Tianjun et al. [19] used OGFC as the parent structure of water-retaining pavement, and proposed a certain theoretical method for water-filled pavement construction technology and grouting quality of water-retaining pavement retaining material. In research on reducing cement cracks, Golewski [20,21] studied the microcracks of cement materials with active mineral additives (fly ash), and obtained the optimum amount of fly ash to improve the durability of cement. Ma et al. [22] studied the effect of SAP on the drying shrinkage, pore structure and permeability of high-performance cement-based materials, and revealed the regulatory effect of SAP on water.

Previously, water-retaining pavement material based on super absorbent polymer (SAP) mortar has been studied, and its water retention performance was mainly provided by SAP. Water absorption capacity of SAP can reach several hundred times that of its own weight. At the same time, it absorbs water quickly and has good water retention performance. Most importantly, SAP can be used as an internal curing agent for cementitious materials. Based on the optimal mix ratio of SAP water-retentive mortar, the mixing ratio of large pore asphalt mixture was determined. Then the large pore structure was filled with water-retentive mortar to prepare a water-retentive asphalt mixture. Finally the road performance, water retention performance and cooling performance were evaluated.

## 2. Experiment

### 2.1. Materials

#### 2.1.1. Cement

The cement used in the study was conventional Portland cement (P·O 42.5 according to Chinese standard (JTG/T F30-2014 [23]). The chemical components and physical properties of it are shown in Table 1 and Table 2, respectively.

#### 2.1.2. Fine Sand

SAP water-retaining mortar developed in this study was intended to be used for perfusion, and the excessive particle size of river sand has a great influence on the fluidity of the mortar. Therefore, the fine sand used in the research was a natural river sand with a small particle size. The performance indicators are shown in Table 3.

#### 2.1.3. Fly ash

Studies have shown that the density of fly ash is between 1.77–2.43 g/cm^3^, and the specific surface area is usually within the range of 0.25–0.70 cm^2^/g [24]. In this paper, some performance tests were carried out according to JTG E30-2005 [25]. The chemical components and specifications of the fly ash are shown in Table 4 and Table 5.

#### 2.1.4. SAP

The SAP used in this paper was produced by Yixing Chemical Co., Ltd., Yixing, China. It was a special type of poly (sodium acrylate) with a low degree of crosslinking. The basic properties of SAP are shown in Table 6. The SAP forms a gel like material when in contact with water. The retention of water by this polymer is relatively good even under external pressure. Table 7 shows particle size of SAP. The maximum water absorption rate is dependent on the particle size.

#### 2.1.5. Porous Asphalt Concrete (PAC)

In this study, crushed basalt aggregates and limestone powder were used for fabricating the PAC mixture. The bitumen used was a high-viscosity modified bitumen manufactured by adding 10 wt.% modifier to the Esso-70 matrix asphalt. The tests were carried out according to JTG E20-2011 [26], and the results are shown in Table 8. The gradation of PAC is presented in Table 9. The optimum binder content was found to be 4.9 wt.% and air void content could be as high as 22.5% of the total PAC specimen.

### 2.2. Preparation and Curing Process of Water-Retentive Asphalt Concrete

To prepare the water-retaining mortar, cement, fine sand, fly ash and SAP were first weighed and mixed. Then, a certain amount of water and other ingredients were added. The ingredients were thoroughly mixed until a uniform composition was obtained. Further experiments were conducted to evaluate the workability of the PAC for filling into the voids to form water-retaining asphalt concrete (WRAC). Before the preparation of WRAC samples, water-retentive mortar (WRS) samples were prepared by first by pouring the mortar into standard Marshall samples and then cured at 20 ± 1 °C with relative humidity no less than 90%. At the same time, tests were conducted to evaluate the liquidity of the fresh mortar and the water absorbing capacity, water retention capacity and compressive strength of the hardened WRC. Finally, the water-retaining asphalt concrete (WRAC) was prepared by pouring the freshly mixed mortar into the porous asphalt concrete followed by a period of curing with desired duration. After preparation, the mortar that remained on the surface of the sample was removed by rubbing with rubber.

### 2.3. Testing Methods

The water absorbing capacity, water retention capacity and compressive strength of hardened water-retentive mortar; liquidity of the fresh mortar; as well as the rutting resistance, moisture susceptibility, low temperature bending resistance, surface slip resistance and cooling effect of water-retentive asphalt concrete were tested using at least three replicate specimens.

#### 2.3.1. Water Absorbing and Retention Capacity

Specimens of 40 mm × 40 mm × 160 mm dimensions were used for water absorbing and retention capacity testing. After the specimen was for 7 days, it was placed in oven at 60 °C until it reached a constant weight. The mass (*W*_0_) of the sample was then weighed when it cooled to the room temperature. After that, the specimens were immersed in water. The weight (*W_n_*) of the wet samples was then weighted after soaking in water for 2, 24 and 48 h. The wet samples removed from water were wiped with lint-free cloth until no water dripped from the sample. The water absorption rate of the water-retentive mortar was calculated using Equation (1).

(1) $$Q=\frac{W_{n}-W_{0}}{W_{0}}$$

where *Q* is the water absorption rate; n = 2 h, 24 h, 48 h.

The water retention performance of the water-retaining mortar was expressed in terms of the mass ratio of the remaining water. After the specimen reached the curing days, it was immersed in water completely.

The specimen was then taken out when the specimen reached the water absorption balance. The wet sample was then wiped dry with a lint-free cotton towel and weighed (m_0_). The specimen was then placed in a blast oven set at 60 °C, and weighed at a frequency of every 2 h for 24 h. The water retention rate of the water-retentive mortar is calculated using Equation (2).

(2) $$R=\frac{m_{t}-W_{0}}{m_{0}-W_{0}}$$

where *R* is the water retention rate (retained water as a percentage of total water).

The reported water absorption and retention results were the arithmetic mean of three individual results.

#### 2.3.2. Liquidity

In order to meet the construction requirements, the water-retentive mortar needs to have a certain fluidity to ensure that it can penetrate into the voids of the porous asphalt concrete. The efflux time is used as an indicator for evaluating the liquidity of the mortar. The shorter the effluent time, the better the processability of the mortar. The device (NLD-3, Kedali Testing Instrument Co., Ltd., Cangzhou, China) used to test the fluidity was a flow cone/funnel (T0507-2017 [25]). In the experiment, the flow cone was fixed and leveled to ensure that it was perpendicular to the ground. The inside of the cone was first wetted with water with the outlet of the discharge tube closed. Then 1725 ± 5 mL of water was introduced into the cone and the horizontal position recorded. The timing was started when the discharge nozzle was released. Timing stopped when the discharged water first appeared intermittent. The water-retentive mortar was thoroughly mixed and quickly poured into the cone after the inner wall of the cone was wetted. After the liquid level reached the previously marked position, the same operation as before was started. Three samples were used for the liquidity test and averaged.

#### 2.3.3. Compressive Strength and Flexural Strength

The water-retaining mortar test specimens with dimensions 40 mm × 40 mm × 160 mm were used for the compressive strength and flexural strength based on the T0506-2005 [25]. Three samples were used for the compressive strength test, and six samples were used for the flexural strength.

#### 2.3.4. Rutting Resistance

The test reference T0719-2011 [26] was made of a 300 mm × 300 mm × 50 mm rutting sample with a wheel mill. The rutting samples were carried out at 60 ± 1 °C.

#### 2.3.5. Moisture Susceptibility

The freeze-thaw split test (T0729-2000 [26]) measured the strength ratio of the sample before and after water damage. First, the four test pieces previously vacuumed for 15 min were placed in water at normal pressure for 0.5 h. Then they were put in a plastic bag filled with 10 mL water. The bag was then stored in a refrigerator for 12 h at −18 °C. In the next step, these pieces were taken out and placed in a water bath for 12 h at 60 °C. This whole procedure constituted a freeze-thaw cycle. The remaining samples were the control group and did not undergo the freeze-thaw cycle. The split test was carried out at a loading speed of 50 mm/min and the maximum load was recorded. Retained Stability (RS) and Tensile Strength Ratio (TSR) are key evaluation indexes.

The freeze-thaw cycle test was carried out to study its effect on the void structure of the asphalt mixture. Each freeze-thaw cycle is the same as described above, and the number of freeze-thaw cycles are: 0 times, 1 time, 5 times, 10 times, 15 times [27]. Five samples were tested in a freeze-thaw cycle. The rate of decline in splitting strength can be evaluated, which was calculated using Equation (3).

(3) $$T=\frac{P_{n}-P_{0}}{P_{0}}$$

where *T* is the water retention rate; P_0_ is the splitting strength without freeze-thaw; P_n_ is the splitting strength of the freeze-thaw cycle n times; n = 0, 1, 5, 10 and15 cycles.

#### 2.3.6. Low Temperature Bending Resistance

The low temperature bending test of the asphalt mixture can be used to evaluate the bending strength of the mixture, the strain at break and the bending stiffness modulus at the time of failure. According to T0715-2011 [26], the water-retaining asphalt mixture rutting sample was cut into a trabecular beam of 30 mm × 35 mm × 250 mm. The MTS test machine (CMT5105, Meters Industrial Systems (China) Co., Ltd., Shenzhen, China) was used, the test temperature was −10 °C and the loading rate was 5 mm/min.

#### 2.3.7. Cooling Effect

**Figure 1:**
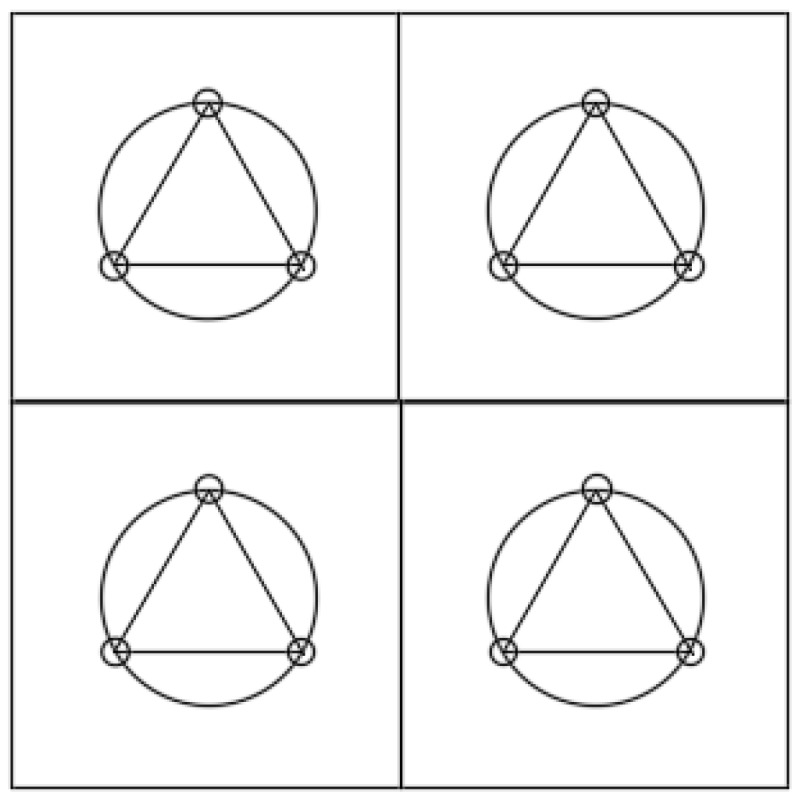
Drilling diagram of sample.

**Figure 2:**
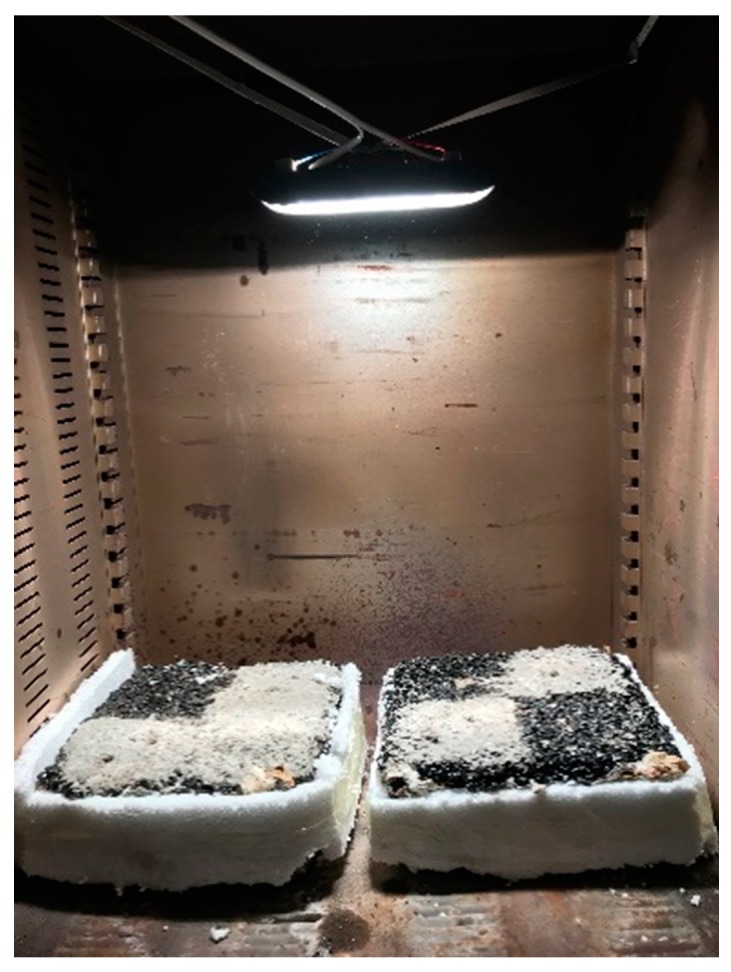
Cooling test site.

The evaluation of the cooling effect provided by the water-retentive asphalt concrete was carried out indoors. Each specimen (300 mm × 300 mm × 50 mm) was divided into four equal portions and filled with water-retentive mortar with optimal SAP dosage. In the center of each aliquot (seen in Figure 1), a circle was drawn with a radius of 40 mm. The circle was then divided into three equal parts, and frilled with a depth around 2 cm to fit the electronic thermometer probe. The surface temperature was measured using an infrared thermometer. The home designed test instrument was shown in Figure 2. The heating lamp that simulates the sunlight was placed 20 cm above the specimens, which ensured that the irradiation was perpendicular to the specimen. After the specimen reaching the curing age was saturated with water, it was placed in an oven. Below the specimen, a common rutting specimen was placed to simulate the underlying layer of the actual pavement; the hole was filled with foam to prevent the lamp illuminating directly. The specimen was surrounded by styrene foam plate for heat preservation. After the heat lamp was turned on, the initial temperature of the deep layer and the surface was read. Then the deep layer and surface temperatures were recorded every 2 h for 8 h. The test was repeated another four times after the sample had cooled to room temperature.

### 2.4. Microscopic Analysis

In the case of conventional drying, SAP is small and is not easily detectable under microscopic conditions when mixed with cement. Some scholars have indirectly proved that SAP is caused by observing large pores [28,29], but the formation of pores is not necessarily due to the loss of water from SAP. Therefore, this paper directly observed SAP through a different method: the broken sample particles were saturated with water and placed in a freeze dryer for 48 h, the temperature was set to −40 °C, the SAP in the sample was kept in the volume after being saturated with water, and then samples were performed using SEM (Hitachi S-4800, Tokyo, Japan). At desired locations, an energy dispersive spectrometer (EDS, X-Max50, Oxford Instrument Technology Co., Ltd., Oxford, UK) was used to reveal the elemental composition of the material and analyze the content.

## 3. Results and Discussion

### 3.1. Preparation of Water-Retaining Mortar

#### 3.1.1. Compressive Strength and Flexural Strength

The mortar sample of the single water-absorbent resin (SAP) is numbered in the form of SX-ZY (abbreviated as XZY in the Table 10), where S represents SAP, X represents the water absorption percentage of SAP, Z represents the type of SAP (A, B, C, and D), and Y represents volumetric content of the water-retentive mortar. For example, 3A7 represents the volume amount of the A-type SAP having a water absorption percentage of 30% was 70%. The composition of the base mortar was 225 g of water, 450 g of cement, 1350 g of sand, and 45 g of fly ash.

**Figure 3:**
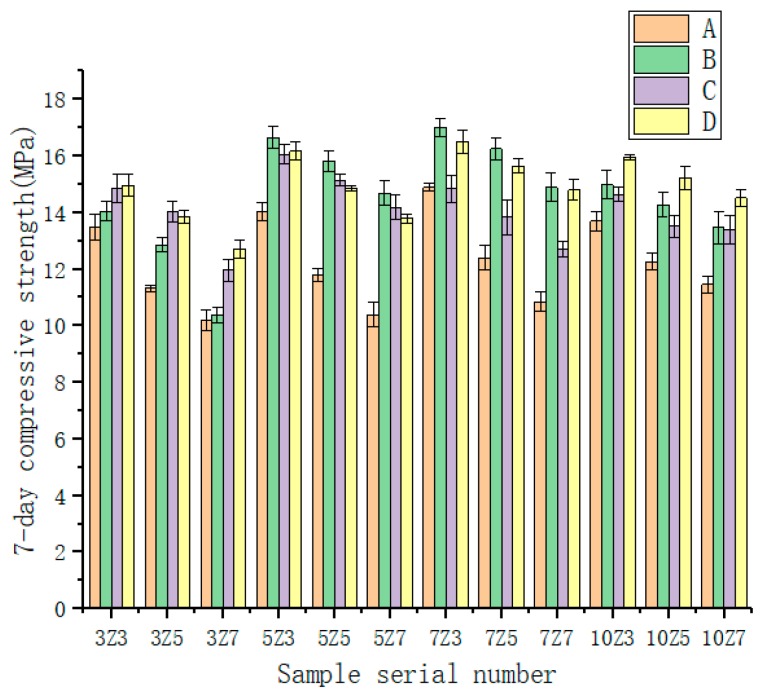
Compressive strength of water-retentive mortar in different SAP types.

**Figure 4:**
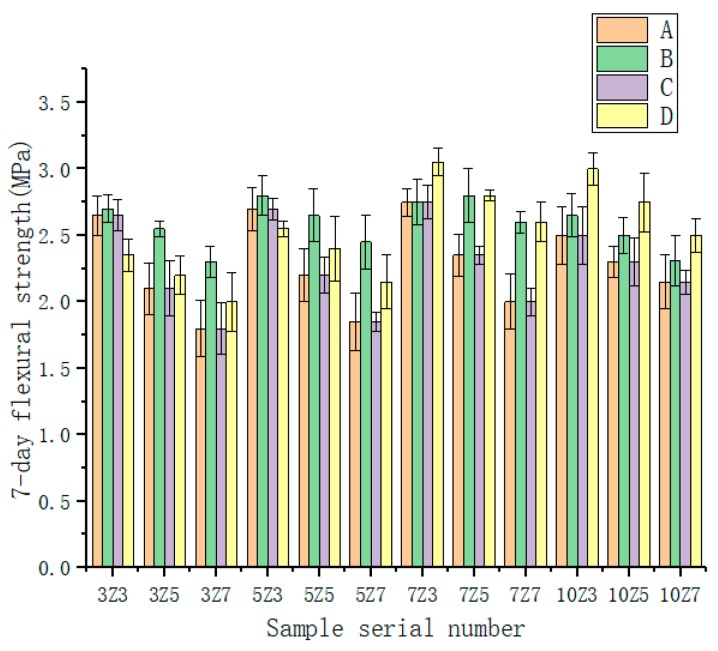
Flexural strength of water-retentive mortar in different SAP types.

Twelve kinds of different water absorption rates and different amounts of SAP were used to test the water-retentive mortar to determine the best mix ratio. It can be seen from Figure 3 and Figure 4 that the 7 d flexural strength of the water-retentive mortar with SAP was above 2.0 MPa, and the 7 d compressive strength was obviously above 10.0 MPa, which meets road design requirements [23]. When the water absorption rate of SAP was the same, the 7 d flexural strength and 7 d compressive strength of the water-retentive mortar gradually decreased with the increase of the water-absorbent resin content. It showed that if the amount of SAP was increased, the strength would be affected adversely, and the effect on 7 d compressive strength was the most significant, with a drop of about 3.0–4.0 MPa. The main reason was that with the increase of SAP content, the water content would increase, although more water would promote the hydration reaction of the cement, but the water supplied by SAP in different mixing ratios exceeded the moisture required for the complete hydration of the cement. The moisture exceeded the moisture required for the complete hydration of the cement. After the water-retaining mortar sample was cured for 7 days, as the moisture in the SAP partially evaporated and reacted, more holes were formed in the water-retentive mortar, and the strength of the water-retentive mortar would decrease as a result. When the amount of SAP was kept constant, with the increase of SAP water absorption rate, the change law of the strength of water-retentive mortar was generally a trend of increasing first and then decreasing, but there was no large difference.

The strength of the water-retaining mortar can meet the requirements. With almost equal proportion of water absorption rate, high strength and economical mixing ratios were selected for as much as possible. Therefore, the initial choice of four kinds of SAP mixing ratios were S50-A-50 and S70-A-50, S50-B-30 and S70-B-50, S50-C-30 and S50-C-70, S50-D-50 and S70-D-50.

#### 3.1.2. Liquidity

**Figure 5:**
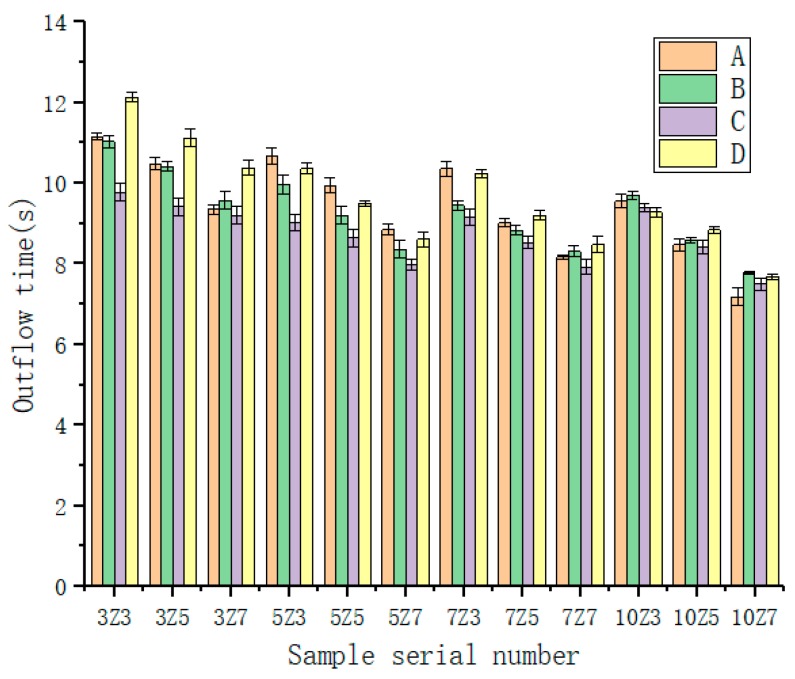
Liquidity of water-retentive mortar in different SAP types.

It can be concluded from Figure 5 that when the water absorption rate of SAP was kept the same, the fluidity of the water-retaining mortar gradually reduced as the SAP content increased. Higher amounts of SAP provided more moisture, which made the water-retentive mortar more fluid. However, while the water absorption rate was also significantly increased, the strength of the water-retaining mortar was negatively affected. The greater amount of SAP content included, after drying the water-retentive mortar, the more water will be absorbed. At the same time, when the amount of SAP was constant, the fluidity of the water-retentive mortar was gradually reduced as the water absorption rate of the water-retentive mortar increased. Mainly because the greater the water absorption of SAP, the more water is supplied, and the better the fluidity of the water-retentive mortar would be. The water absorption rate of the water-retentive mortar first increases and then decreases, indicating that the water absorption rate of SAP is not as high as possible. The fluidity was less than 12 s, which meets the requirements [1], but low fluidity is beneficial to the mortar filling porous asphalt concrete. Combined with the initial selection, meeting the requirements of liquidity and economy, the final four mixing ratios selected were S50-A-50, S70-B-50, S50-C-30 and S50-D-50.

### 3.2. Screening of Water-Retentive Mortar

According to the mechanical strength, SAP content and fluidity, the optimal mix ratio of the four types of SAP water-retentive mortar is shown in Table 11.

#### 3.2.1. Water Absorbing and Retention Capacity

**Figure 6:**
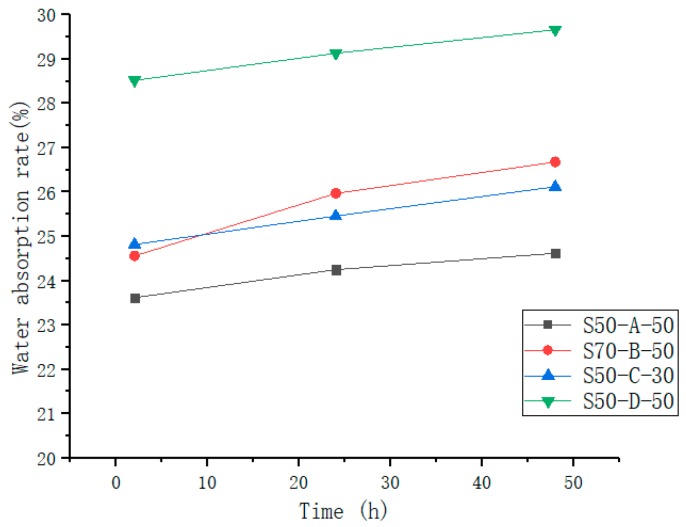
Water absorption rate of water-retentive mortar at different times.

The water-retaining material should have good water absorption and water retention, which can ensure that the road surface can quickly absorb water under the condition of rain, preventing the forming of puddles or water rafts on the road surface, and improving the ability of the road surface to cool down. It can be seen intuitively from Figure 6 that the water absorption of S50-D-50 was significantly higher than other mix ratios, and the water absorption of the water-retentive mortar increases with time. The increase in water absorption between 2 h and 24 h was substantially the same as the increase between 24 h and 48 h. The water-retaining mortar could absorb more water in a short time and continuously absorb water for a long time, indicating that it could adapt to short-term precipitation and watering, and absorb enough water during long-term precipitation. From the test results, S50-D-50 showed the best water absorption effect.

**Figure 7:**
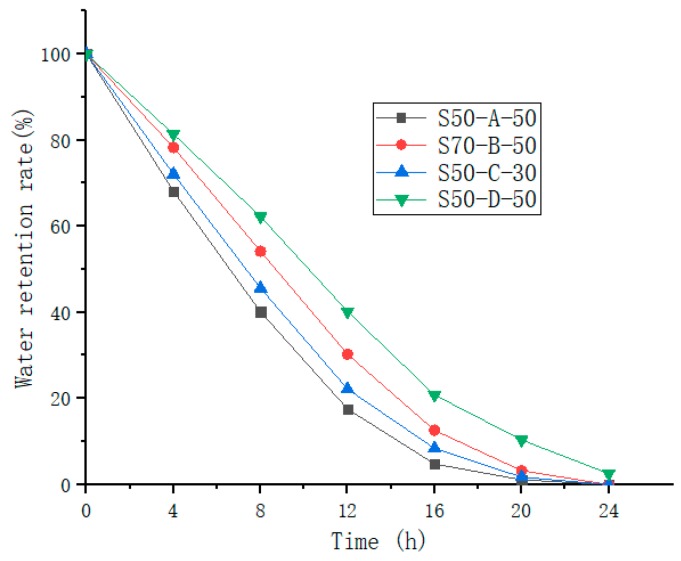
Water retention rate of water-retentive mortar at different times.

It can be seen from Figure 7 that with the increase of time, the water retention rate of the water-retaining mortar was decreased, and the downward trend was obvious. After 24 h, the water retention rates of S50-A-50, S70-B-50, S50-C-30 dropped to 0%, and only S60-D-50 had 2.56% remaining. In practice, the time of the road surface under such high temperature conditions will not be too long, so the number of days of water retention can be extended. In summary, the final mix ratio of the water-retaining mortar was water-absorbing resin with D-type SAP having a water absorption of 50%, and a volume amount of 50%.

#### 3.2.2. Microscopic Analysis

In order to observe the microscopic morphology of the particles, the interfacial state of the water-retentive mortar was studied by collecting SEM images with magnification in the range of 5000–10,000 times. Then, the elemental composition and content of the granules were obtained by EDS to determine the composition of the material.

**Figure 8:**
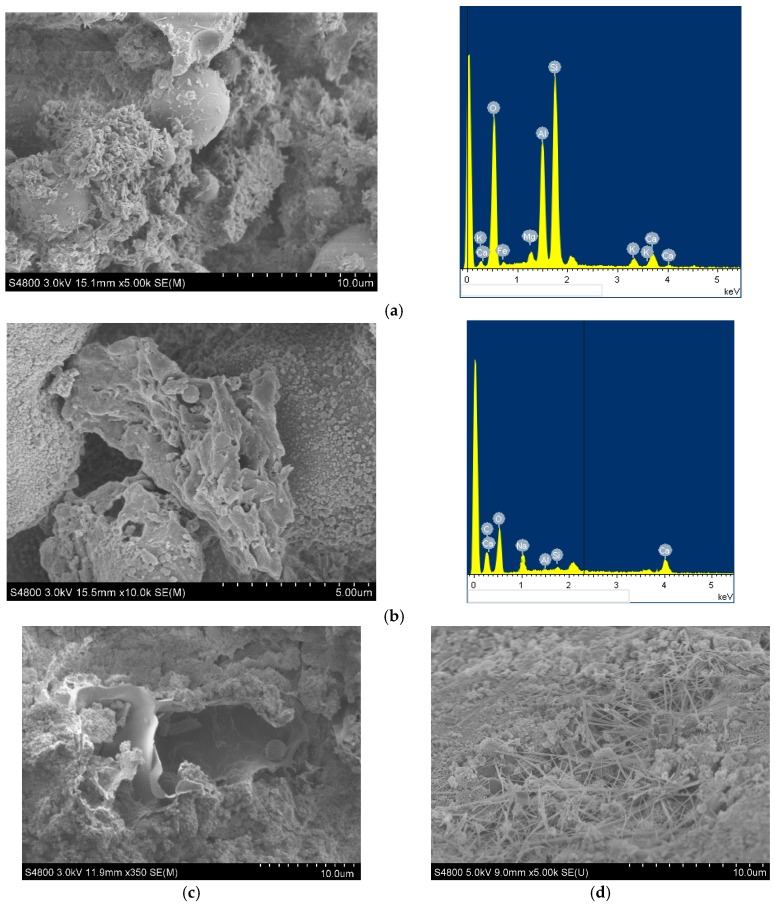
SEM images. (**a**) Fly ash; (**b**) SAP; (**c**) hole formed by dry SAP; (**d**) AFt and Ca(OH)_2_.

Abundant hydration products, such as calcium silicate hydrates (C-S-H) and ettringite (AFt), can be clearly seen in Figure 8. It can be also seen in Figure 8a that the hydration of fly ash, 66 μm in diameter, took place under the action of alkali activation. The sample was freeze-dried so that the volume of the SAP did not change much after water loss, as shown in Figure 8b, and elemental analysis by energy spectrometer revealed that the elemental composition was similar, and the diameter was only 6 μm. Generally, the dried SAP has a particle size of 3–38 μm and rapidly expands to 200–600 μm after being saturated with water [30]. Some of the dried SAP particles formed grooves (Figure 8c) that collapse to form a film-like stacked structure [31,32]. After the release of water, SAP formed uniformly distributed unconnected micropores in the mortar, which could buffer the forces caused by freezing of water, and were beneficial to reduce the permeability and pore connectivity of the mortar. A large amount of ettringite crystals existed between the film and the pore walls (Figure 8d), and the Ca(OH)_2_ and AFt were distributed in a discontinuous three-dimensional structure. The water–cement ratio near the SAP particles is relatively large, which promotes the mass production of ettringite, and the pores left after the formation of SAP water loss collapse provide sufficient space for the growth of ettringite, so the ettringite grows densely near the SAP film and the microporous wall, and is filled to some extent by the pores formed by the SAP, significantly inhibiting plastic shrinkage and microcracks.

### 3.3. Properties of Water-Retaining Asphalt Concrete

**Figure 9:**
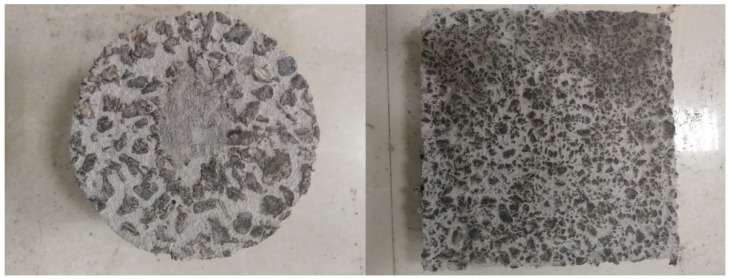
Samples after pouring water-retentive mortar (the left is Φ101.6 mm × 63.5 mm Marshall sample, and the right is 300 mm × 300 mm × 50 mm rutting sample).

The S50-A-50, S70-B-50, S50-C-30 and S50-D-50 types of water-retentive mortar were prepared for mixture performance test. The water-retaining mortar was poured into the air void of the porous asphalt concrete through a vibrating table. After 28 days of solidification, the water-retentive asphalt concrete sample was tested and evaluated according to experiments on the rutting resistance, moisture susceptibility, low temperature bending resistance, surface slip resistance and cooling effect. Figure 9 shows two examples of the Marshall and rutting samples of water-retentive asphalt concrete. In order to compare with water-retentive asphalt concrete, the same test was conducted on the porous asphalt concrete samples.

#### 3.3.1. Rutting Resistance

Wheel tracking test was used to evaluate the rutting resistance of water-retentive asphalt concrete and PAC. Table 12 shows the test results with index of Dynamic Stability (DS), indicating that all mixtures showed good high temperature stability. The DSs of S50-A-50, S70-B-50, S50-C-30 and S50-D-50 water-retentive asphalt concrete were 9336, 10,024, 9658 and 10,552 times/mm, respectively, nearly twice the DS of PAC (5374 times/mm). The aggregates of PAC were in contact with each other in the form of dots, and the embedded structure was poor. However, high viscosity asphalt modifier added during the mixing process, which significantly increases the viscosity of the asphalt binder, caused the high temperature stability of PAC. For water-retentive asphalt concrete, in addition to the effect of high-viscosity asphalt modifier, the infused water-retentive mortar was filled into the gap between the aggregates, and the structure of the mixture was transformed into a “skeleton-densified” structure, which supports the aggregate. The ability to resist deformation has been greatly improved. Therefore, the aggregate in the water-retaining asphalt concrete was not easily moved by exposure to high temperature. Furthermore, the SAP particle size and water absorption rate have different effects. The smaller the particle size, the larger the specific surface area, the stronger the adhesion and water absorption capacity. Therefore, the water-retentive asphalt concrete had the more anti-rutting ability than the PAC.

#### 3.3.2. Moisture Susceptibility

Marshall immersion test and freeze-thaw split test were used to evaluate the moisture susceptibility of water-retentive asphalt concrete and PAC. Water-retentive asphalt concrete has a much smaller air void than PAC due to the hardened water retention mortar filled in the air voids, which is beneficial to increasing the overall strength and stability.

As shown in Table 13, water-retentive asphalt mixture and PAC showed good sensitivity to moisture. The Marshall stability and retained stability of the water-retentive asphalt mixture were higher than that of the PAC, and the retained stability reached 88% which was higher than the standard value (RS ≥ 85%). It showed that the filling of the water-retaining mortar improved the water stability of the large pore asphalt pavement. Furthermore, it can be seen that the freeze-thaw splitting strength of the water-retaining asphalt mixture met the requirements of the specification (TSR ≥ 80%). The filling of the water-retaining mortar did not reduce the TSR of the asphalt mixture, indicating that the durability of the water-retaining asphalt concrete was not significantly reduced, but had a certain improvement.

**Figure 10:**
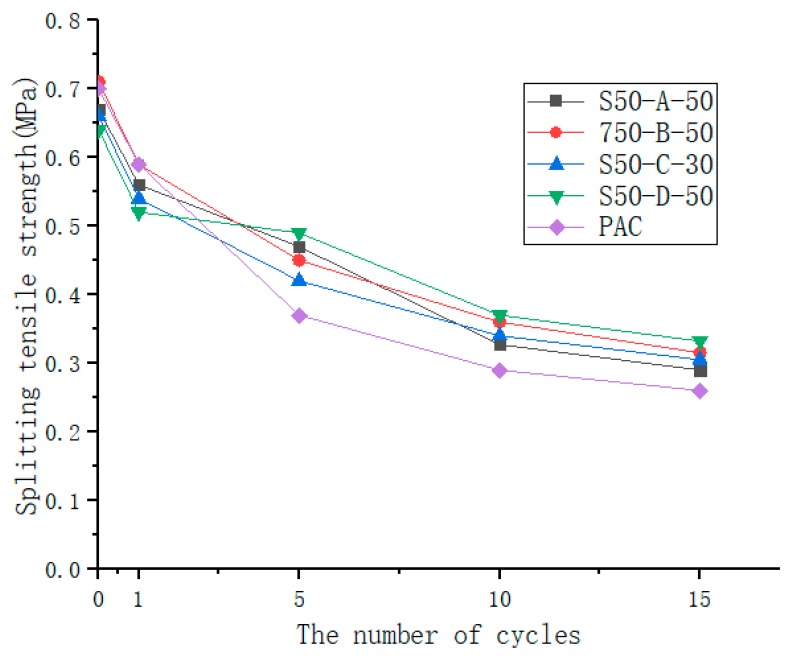
Splitting tensile strength after freeze-thaw cycle.

**Figure 11:**
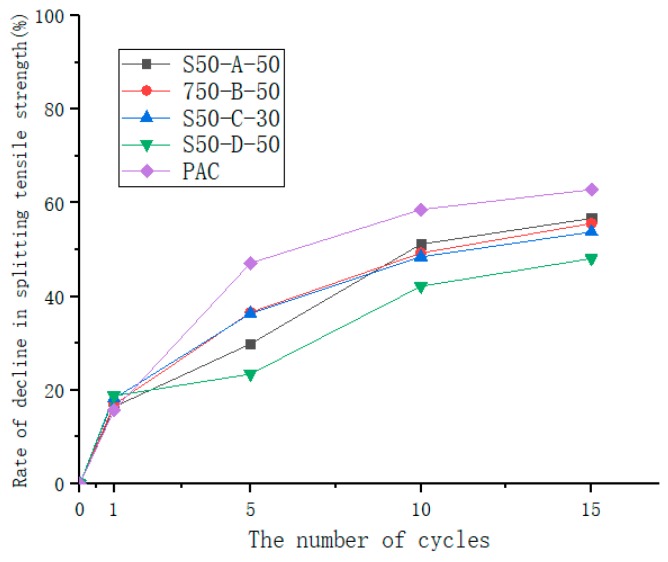
Rate of decline in splitting tensile strength after freeze-thaw cycle.

As shown in Figure 10, splitting tensile strength decreased with the increase of the number of freeze-thaw cycles. The splitting tensile strength of the mixture decreased significantly during the first few freeze-thaw cycles. After about 10 freeze-thaw cycles, the rate of change in splitting tensile strength slowed and the trend gradually became flat. It can be seen from Figure 11 that SAP mortar is effective for improving the splitting strength. After 15 freeze-thaw cycles, the rate of decline in the splitting strength of PAC was 61.9%, while the drop rate of SAP was 48.1%–55.3%, which was less susceptible to water damage than PAC. The splitting tensile strength of asphalt mixture is mainly composed of the skeleton action of coarse aggregate, the bonding action of asphalt and SAP cement mortar. Under the action of repeated saturated freezing and thawing cycles, the internal voids of the specimens will enter the water, and the free water and the crystal water in the SAP will increase in volume after freezing, resulting in a large expansion force. The adhesion of asphalt to aggregates decays faster, so that the splitting tensile strength of the mixture decreases rapidly during the first few freeze-thaw cycles. As the number of freeze-thaw cycles increases, the internal pores of the mixture become larger, the structure will become loose, and the external moisture will easily enter the interior of the asphalt membrane, weakening the adhesion between the asphalt membrane and the aggregate. The contribution of the splitting tensile strength is reduced, but the cement mortar has a certain strength, and the asphalt and aggregate are wrapped to reduce the adhesion attenuation.

#### 3.3.3. Low Temperature Bending Resistance

According to the asphalt mixture bending test procedure, the stiffness modulus value can be used to characterize the crack resistance of the mixture. The smaller the modulus value, the greater the energy required to break the material, and the better the crack resistance of the mixture. The bending strain reflects the low temperature resistance of the asphalt mixture. The test results are shown in Table 14. It can be seen that the water-retaining asphalt mixture has a lower bending strength than PAC, of which S70-B-50 was 6.48 MPa. The bending strain of S50-D-50 was 3383 MPa. It was found that when the bending strain was larger, the stiffness modulus of water-retentive asphalt mixture was much smaller. It can be seen that the filling of the water-retentive mortar improved the low-temperature performance of the large-pore asphalt pavement. The main reason is that PAC was a typical mixture with a skeleton-void structure which resulted in a relatively small bond area of aggregated particles and the macroscopic and microscopic texture of the water-retentive asphalt mixture made it easier to generate stress concentrations [1].

### 3.4. Cooling Effect

**Figure 12:**
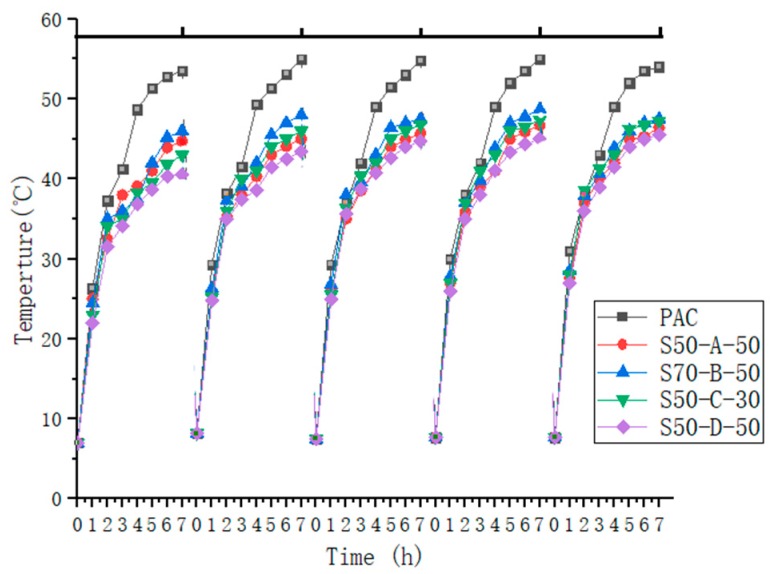
The surface temperature of the five mixtures.

**Figure 13:**
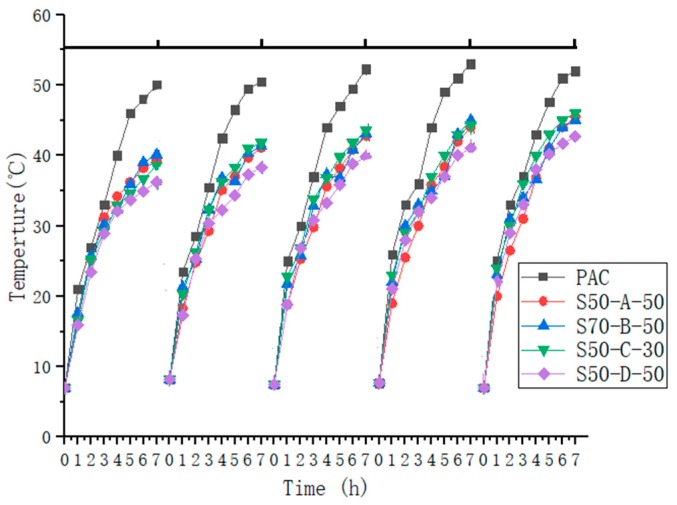
The internal temperature of the five mixtures.

One of the most important characteristics of water-retaining pavement is the cooling effect. The paper conducted the experiment indoors from 9:00 to 17:00 every day to simulate the daytime. It can be seen from Figure 12 and Figure 13 that the surface temperature of the five mixtures was higher than the temperature inside the mixtures. Within 3 h exposure to heating lamp, the surface temperature difference between the five samples was small, due to containing a certain amount of water. Then, as the time went on, the temperature difference between PAC and SAP water-retaining asphalt mixture increased first and then decreased. The cooling effect of S50-D-50 water-retaining asphalt mixture was better than that of the other three SAP water-retaining asphalt mixtures, owing to the small particle size of S50-D-50, the water storage capacity was strong, the surface and internal temperature difference with PAC reached 13.1 °C and 14.3 °C, respectively. The reason can be explained by considering two aspects. On the one hand, the moisture content of the water-retaining asphalt mixture and the evaporation of water takes away most of the heat. On the other hand, the overall color of the asphalt mixture was black, and the asphalt was a highly heat-absorbing material. If the temperature cannot be effectively lowered, the temperature of the asphalt pavement will be much higher than the atmospheric temperature, while the color of the water-retaining mortar is white, which reduces the absorption rate of solar radiation. At a depth of 2 cm, the increase in temperature is mainly caused by heat conduction, while the heat transfer of the mortar is slower, and the SAP in the upper mortar absorbs from the lower layer after losing water. Therefore the temperature inside the water-retentive asphalt mixture is lower than the temperature inside the PAC.

However, after five cycles, it can be found that the temperature difference between PAC and SAP water-retentive asphalt mixture was reduced, but the reduction range was decreased each cycle and stabilized. The surface and internal temperature differences were 4.3–8.2 °C and 6.1–9.8 °C after the fifth time, respectively. Due to SAP adherence to pollutants (such as dust) before each immersion, it reduces the ability to store water repeatedly, but the water absorption capacity will gradually stabilize and still have the effect of cooling.

**Figure 14:**
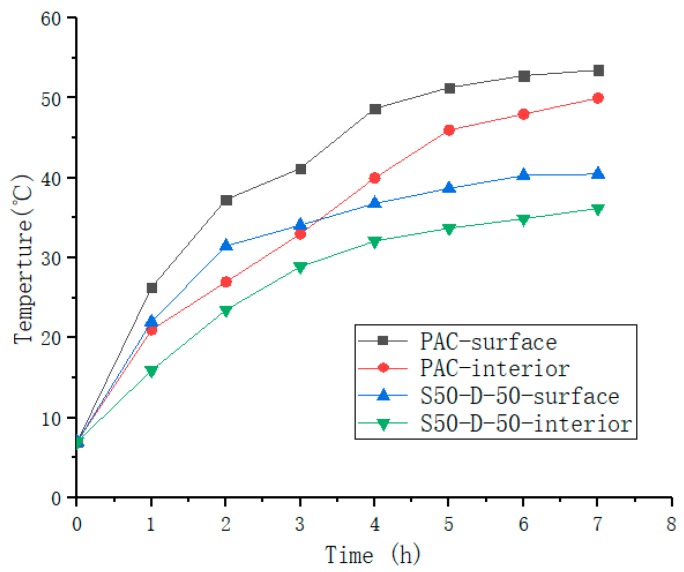
The surface and internal temperature in the first cycle.

Since the comparison of S50-D-50 with PAC is representative, a cycle is listed separately for analysis (Figure 14). The results demonstrated that as discussed previously, the surface temperatures of the two mixtures were higher than the internal temperatures, the difference in the former temperature accelerated, and the latter difference of temperature was gradually reduced, associated with the accelerated loss of SAP water. The surface temperature of the S50-D-50 was higher than the internal temperature of the PAC due to direct illumination, and the temperature growth of the SAP water retention fell. It appears that it can reduce the road temperature, improve the high temperature stability of the road surface, absorb and preserve rainwater, and slow down the urban heat island effect. This material is suitable for the temperate monsoon climate and the subtropical monsoon climate, and has important economic impact potential.

## 4. Conclusions

SAP water-retaining asphalt concrete can meet the requirements of pavement performance, while reducing the heat island effect, dust pollution, and their negative impacts on people and the environment. Based on the obtained test results, the following conclusions can be drawn:

(1) Considering the optimal water absorption rate and water retention rate, high amounts of SAP will provide more moisture, which will make the water retention mortar more fluid. The final mix ratio of the water retention mortar was selected as a D-type SAP with a water absorption of 50%, and a volume amount of 50%.
(2) The water–cement ratio near the SAP particles is relatively large, which promotes the mass production of ettringite, and the pores left after the formation of SAP water loss collapse provide sufficient space for the growth of ettringite.
(3) The moisture susceptibility (RS ≥ 88.2%, TSR ≥ 81.8%), rutting resistance (9336–10,552 times/mm) and low temperature crack resistance (3383–3621 MPa) of water-retaining asphalt concrete were superior to porous asphalt concrete.
(4) The absorption of heat after SAP lost water made the cooling effect (10–12 ℃) of water-retaining asphalt concrete obvious.

## Figures and Tables

**Table 1:** Chemical and phase analysis of the cement (wt.%).

**Chemical Components**	**SiO_2_**	**Al_2_O_3_**	**CaO**	**Fe_2_O_3_**	**MgO**	**K_2_O**	**Na_2_O**	**SO_3_**
The cement	22.41	5.84	62.72	3.08	1.98	0.39	0.12	1.99
**Phase Components**	**C_3_S**		**C_2_S**		**C_3_A**		**C_4_AF**	
The cement	55.4		26.3		6.5		11.8	

**Table 2:** Physical properties of the cement.

Density (g/cm^3^)	Setting Time (min)		Flexural Strength (MPa)		Compressive Strength (MPa)	
	Initial	Final	7 d	28 d	7 d	28 d
3.11	150	275	5.07	7.11	26.27	46.37

**Table 3:** Basic performance of fine sand.

**Property**	**Test Result**	**Property**	**Test Result**
Mud content (%)	0.65	Maximum Particle Size (mm)	1.18
**Screening test**			
**Sieve Size (mm)**	**Scoring Rate (%)**	**Cumulative Screening Rate (%)**	**Through Rate (%)**
0.6	1.1	1.1	98.9
0.3	5.9	7.0	93.0
0.15	37.9	44.9	55.1
0.075	52.1	97.0	3.0

**Table 4:** Chemical analysis of fly ash (wt.%).

Components	SiO_2_	Al_2_O_3_	Fe_2_O_3_	CaO	MgO	SO_3_	Na_2_O	K_2_O	P_2_O_5_
The fly ash	44.08	41.53	3.98	5.80	0.58	0.97	0.82	1.14	1.10

**Table 5:** Basic performance of fly ash.

Properties	Test Result	Criteria
Fineness (0.045 mm square hole sieve) (%)	18.6	≤20
Water demand ratio (%)	95	≤105
Loss on ignition (%)	5	≤8
Water content (%)	0.4	≤1.0
SO_3_ content (%)	2.5	≤3.0
Water absorption rate (%)	95	-
Porosity (%)	41	-

**Table 6:** Basic properties of the super absorbent polymer (SAP).

Properties	Test Result
Bulk density	0.68 g/cm^3^
PH	6.7
Volatile content	5.2%
Anti-caking	Not easy to agglomerate

**Table 7:** Particle size of the SAP.

Types of SAP	Particle Size (mesh)
A	40–60
B	60–100
C	100–200
D	200–400

**Table 8:** Technical index of high viscosity modified asphalt.

Properties		Criteria	Test Result
Penetration (25 °C, 100 g, 5 s) (0.1 mm)		≥40	58
Softening point (°C)		≥80	86.4
Ductility of 5 °C (cm)		≥20	41
Film oven heating test (163 °C, 5 h)	Quality loss (%)	≤0.6	0.19
	Penetration ratio (%)	≥65	81
	Ductility of 5 °C (cm)	≥30	47.5
Rotational viscosity of 60 °C (Pa·s)		≥20,000	32,426

**Table 9:** Gradation of PAC-13 for testing.

Sieve Size (mm)	16.0	13.2	9.5	4.75	2.36	1.18	0.6	0.3	0.15	0.075
Through rate (%)	100	93.3	74.8	48.7	29.3	18.7	14.3	9.9	6.7	3.2

**Table 10:** The number of SAP mortar samples.

Water Absorption Percentage of SAP (%)	Volumetric Content of the Water-Retentive Mortar (%)	Abbreviation
30	30	S30-Z-30(3Z3)
30	50	S30-Z-50(3Z5)
30	70	S30-Z-70(3Z7)
50	30	S50-Z-30(5Z3)
50	50	S50-Z-50(5Z5)
50	70	S50-Z-70(5Z7)
70	30	S70-Z-30(7Z3)
70	50	S70-Z-50(7Z5)
70	70	S70-Z-70(7Z7)
100	30	S100-Z-30(10Z3)
100	50	S100-Z-50(10Z5)
100	70	S100-Z-70(10Z7)

**Table 11:** The mix ratio of the four types of SAP water-retentive mortar.

Types of SAP	The Mix Ratio
A	S50-A-50
B	S70-B-50
C	S50-C-30
D	S50-D-50

**Table 12:** Dynamic Stability test results.

Types of Asphalt Mixtures	Dynamic Stability (times/mm)
S50-A-50	9336
S70-B-50	10,024
S50-C-30	9658
S50-D-50	10,552
PAC	5374

**Table 13:** Moisture susceptibility test results (one freeze-thaw cycle).

Properties	S50-A-50	S70-B-50	S50-C-30	S50-D-50	PAC
**Marshall stability (kN)**	7.62	7.67	7.51	7.75	7.48
**Retained stability (RS) (%)**	90.4	91.1	88.2	92.5	87.3
**Splitting tensile strength (MPa)**	0.67	0.71	0.66	0.70	0.64
**Splitting tensile strength after freeze-thaw (MPa)**	0.56	0.59	0.54	0.59	0.52
**Tensile Strength Ratio (TSR) (%)**	83.5	83.1	81.8	84.3	80.7

**Table 14:** Low temperature bending test results.

Properties	Bending Strength (MPa)	Bending Strain (10^−3^)	Stiffness Modulus (MPa)
PCA	6.67	1.81	3685
S50-A-50	6.62	1.85	3577
S70-B-50	6.46	1.78	3621
S50-C-30	6.58	1.88	3494
S50-D-50	6.53	1.96	3383
